# Psoriasis risk in patients with diabetic retinopathy: A nationwide population-based study

**DOI:** 10.1038/s41598-018-27147-0

**Published:** 2018-06-14

**Authors:** Ji Hyun Lee, Ju Hee Han, Kyung Do Han, Young Min Park, Jun Young Lee, Yong-Gyu Park, Young Bok Lee

**Affiliations:** 1 Department of Dermatology, College of Medicine, The Catholic University of Korea, Seoul, Republic of Korea; 2 Department of Biostatistics, College of Medicine, The Catholic University of Korea, Seoul, Republic of Korea

## Abstract

Psoriasis is a chronic cutaneous disease known to be related with systemic disease; however, the association between psoriasis and diabetic complications has not been previously reported. Diabetic microvascular complications include diabetic retinopathy (DR), nephropathy, and neuropathy, and overt diabetic nephropathy can lead to the end-stage renal disease (ESRD).The present study investigated the association between psoriasis and non-proliferative DR (NPDR) or proliferative DR (PDR) or ESRD. We analyzed the relationship between diabetic complication and psoriasis using data from the National Health Insurance Service between 2009 and 2015. During a mean follow-up of 5.18 years, 43,792 patients were newly diagnosed with psoriasis. In Cox proportional hazard models, patients with NPDR (hazard ratio [HR] 1.26) had a higher incidence of psoriasis and patients with PDR patients had a higher risk of psoriasis (HR 1.35). ESRD was defined by the ICD-10 code, including chronic kidney disease/renal failure, transplantation, and dialysis. The incidence of psoriasis increased in DR patients with ESRD (HR 2.99, 95% CI 2.49–3.59, p < 0.001) compared to non-DR patients without ESRD. This is the first association study between psoriasis and diabetic complications including DR and ESRD. DR and its severity were related to the onset of psoriasis. In addition, ESRD was related with an increased psoriasis in DR patients.

## Introduction

Psoriasis is a skin disorder with chronicity that is known to have a prevalence of about 2–4%^1^. The trends of prevalence of psoriasis in certain countries are rising^2–4^. Psoriasis is considered to be a systemic disease associated with various comorbidities such as cardiovascular disease, stroke, hypertension, dyslipidemia, diabetes, metabolic syndrome, and obesity^5,6^. Diabetes in patients with psoriasis implies a risk for stroke.

Diabetes mellitus (DM) is also increasing worldwide. According to the World Health Organization’s diabetes fact sheet, approximately 422 million people worldwide have diabetes^7^. Diabetes is also associated with the development of other diseases, and the health burden for diabetes is expected to continually increase. In 1966, Brownstein first suggested that psoriasis was associated with type 2 DM^8^. In a recent meta-analysis, patients with psoriasis were shown to have an increased risk of diabetes, and the pooled OR was 1.76 (95% CI 1.59–1.96) in the random effect model analysis^9^. Although there are some studies on the risk of diabetes in patients with psoriasis, there is little research assessing the risk of psoriasis in patients with DM^10,11^.

Diabetic retinopathy (DR), nephropathy and neuropathy are microvascular complications that are closely related to blood glucose control and are indicators of the disease duration. Overt diabetic nephropathy can lead to end-stage renal disease (ESRD). After 20 years of DM diagnosis, ESRD has been reported to occur up to 17%^12,13^. However, there is little known about the potential effects of DR or ESRD on psoriasis. Therefore, the present study examined the link between DR/ESRD and the psoriasis risk in diabetic patients through a population-based cohort study.

## Methods

### Data source and study population

The National Health Insurance Service (NHIS) database includes the majority of the Korean population. The computerized database of the NHIS includes all claim data contained most medical information related to the patient. A diagnosis was confirmed based on the ICD-10-CM codes^14^. This study was approved by the Institutional Review Board of the Korean National Institute for Bioethics Policy (NHIS-2017-1-004) and the Ethics Committee of Seoul St. Mary’s Hospital, The Catholic University of Korea (KC17ZESI0233) and was conducted per the principles of the Declaration of Helsinki. The Institutional Review Board at the Korea Centers for Disease Control and Prevention approved the protocol. Anonymized and de-identified information was used for analysis, and informed consent was not required.

### Study population

**Figure 1:**
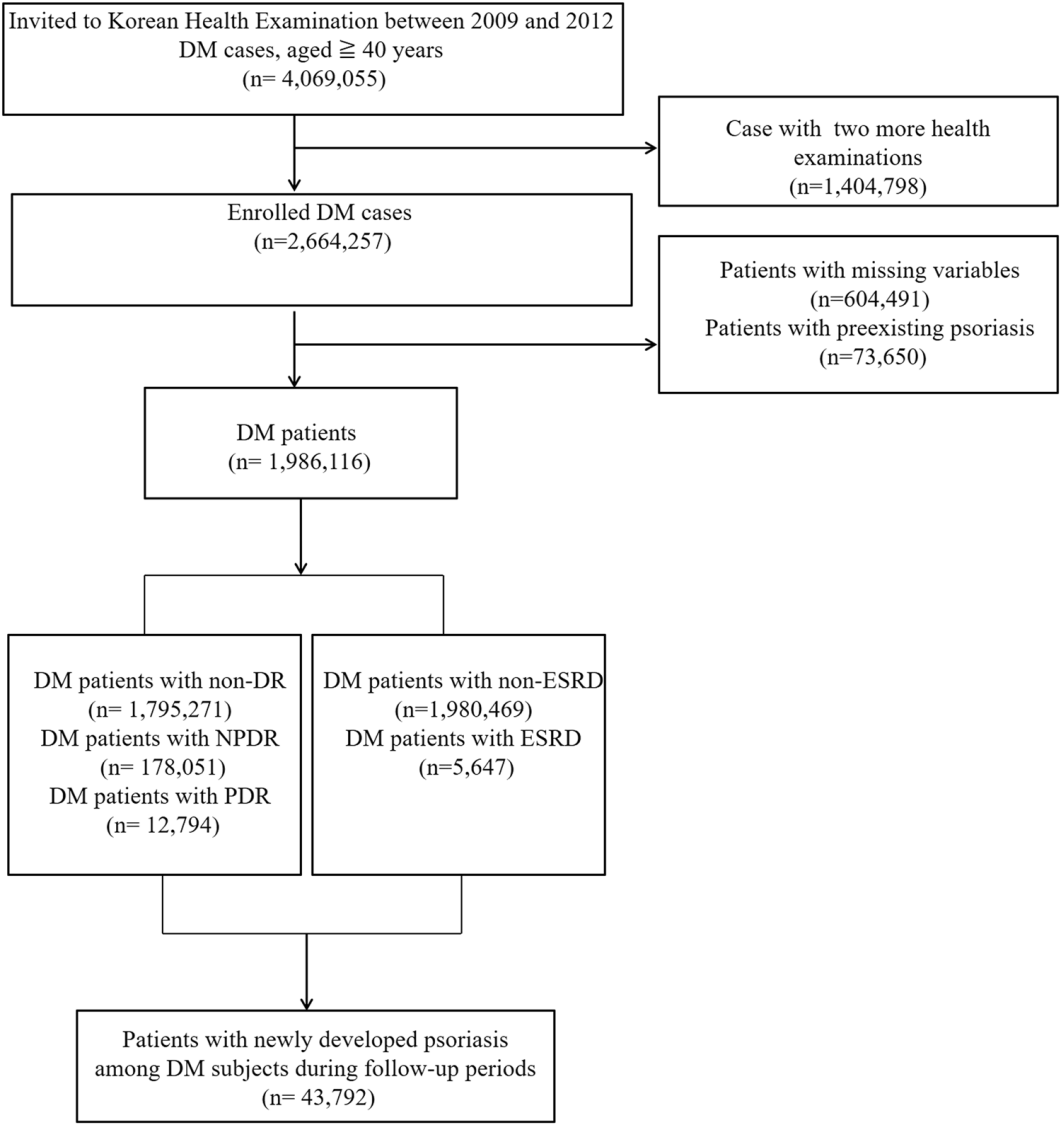
Flow chart of enrolled patients.

From the NHIS data, we enrolled patients aged 40 years or older who underwent health screenings from January 2009 to December 2012. Among these subjects, we focused on patients with type 2 DM (Fig. 1). At that time, enrolled DM patients were linked claim data and confirmed by medical details. In other words, a diagnosis of type 2 diabetes was confirmed by the ICD-10-CM diagnostic code E11-E14 and the prescription of antidiabetic drugs (sulfonylureas, metformin, meglitinides, thiazolidinediones, dipeptidyl peptidase-4 inhibitors, α-glucosidase inhibitors, or insulin)^15^. Patients were also included with type 2 diabetes when they were admitted to the hospital more than once or visited the outpatient clinic more than twice. The baseline (time 0) is the time when diabetes was identified among people who received a health examination between 2009 and 2012.

### Subgroup classification

To closely examine the relationship between DM complication and psoriasis risk, enrolled DM patients during health examination were connected with claim data and subdivided into subgroups based on the presence of NPDR, PDR, and ESRD at baseline. DR was defined as a DR diagnostic code (H360) in type 2 diabetic patients. Proliferative DR (PDR) was defined by the procedure code S5160 for panretinal photocoagulation in patients already diagnosed with DR^15,16^. Therefore, among the DR patients, the remaining patients except PDR patients were defined as non-proliferative DR (NPDR) patients. End stage renal disease (ESRD) was defined by the presence of at least one claim per year under ICD-10 codes N18, N19, Z49, Z905, Z94, and Z992, and procedure codes R3280 (kidney transplantation), O7011–7020 (hemodialysis), and O7017 and O7075 (hemodialysis).

### Incidence of Psoriasis

From January 2009 to December 2012, patients with DM were enrolled from health screening and patients with psoriasis before the enrollment as a DM patient were excluded. Finally, 1,986,116 patients with type 2 diabetes were studied. At that time, patients were connected to the claim data and evaluated for DM complications such as NPDR, PDR, and ESRD, and were classified as subgroups. This study investigated newly diagnosed patients with psoriasis (ICD-10 code, L40) by dermatologists among diabetic patients during follow-up period through the claim data. The primary end point was newly diagnosed psoriasis among registered patients with DM until December, 2015.

### Data and Baseline comorbidities

Enrollees in the National Health Insurance Corporation are recommended to undergo standardized medical examination every two years. Standardized medical examination included measurements of height, weight, waist circumference, and blood pressure (BP) and laboratory tests such as fasting glucose, total cholesterol, HDL, and urinalysis. Past medical history including heart disease and stroke, and health-related behaviors such as smoking, drinking, and physical activity were collected using standardized self-reporting questionnaires. Smoking status was categorized as non-smoker, former-smoker or current smoker. Alcohol drinking status was categorized as non-drinker or mild drinker (<30 g/day), or heavy drinker (≥30 g/day) based on a question regarding the frequency of alcohol consumption per week. Income level was dichotomized at the lowest 20%. The hospitals in which the health examinations were performed were certified by the NHI Service and subject to regular quality control. Baseline characteristics were obtained from the health examination and claim data during the screening period (2009–2012). We observed hypertension and dyslipidemia with the following criteria. Hypertension was defined as (1) one or more claims/year for an antihypertensive prescription under ICD-10 codes I10–I15 or (2) systolic/diastolic BP ≥ 140/90 mmHg L. Dyslipidemia was defined as (1) one or more claims/year for antihyperlipidemic prescription under ICD-10 code E78 or (2) total cholesterol ≥6.21 mmol/L^15^.

### Statistical analysis

Baseline characteristics are expressed as mean with standard deviation or numbers and percentages. The incidence of psoriasis was analyzed by dividing the number of incident cases by the total follow-up period. The incidence of psoriasis is presented as 1000 person-years.

Cox proportional hazards regression analysis was used to investigate the relationship between NPDR or PDR or ESRD and the occurrence of psoriasis. Compared with the control group, HR and 95% CI of each group were calculated. In model 1, age and gender were adjusted as confounding variables. Model 2 adjusted for age, gender, smoking status, alcohol consumption, physical activity, and income level were considered as confounding variables. Additionally, in Model 3, body mass index (BMI), fasting glucose, hypertension, and dyslipidemia were adjusted. In this study, ESRD was considered separately from other complications, and the group was subdivided based on the presence of DR and ESRD.

The cumulative incidence of psoriasis with the presence of NPDR or PDR was presented using the Kaplan-Meier curve. We also performed a log-rank test to analyze differences between groups. Statistical significance was defined as a two-sided P-value lesser than 0.05. All statistical analyses were performed using SAS software (ver. 9.4; SAS Institute, Cary, NC, USA) and R programming (version 3.1.0; The R Foundation for Statistical Computing, Vienna, Austria).

### Ethics

This study was approved by the Institutional Review Board of the Korean National Institute for Bioethics Policy (NHIS-2017-1-004) and the Ethics Committee of Seoul St. Mary’s Hospital, The Catholic University of Korea (KC17ZESI0233) and was conducted per the principles of the Declaration of Helsinki.

## Results

### Baseline characteristics of study population

We identified 1,986,116 patients with type 2 DM (60.19% men). In this study, those with DR accounted for 9.61% of all diabetic patients (190,845 of 1,986,116 patients). Individuals were categorized into the non-DR group (n = 1,795,271), NPDR group (n = 178,051), or PDR group (n = 12,794). Characteristics of subjects are described in Table 1. The male patients were 60.19% in the non-DR group, 47.47% in the NPDR group, and 54.06% in the PDR group. Comorbidities including hypertension, dyslipidemia, stroke and heart disease were observed more frequently in DR group than non-DR group (all, p for trend < 0.001). Hypertension and stroke were observed more common in the PDR group than in the NPDR group.

**Table 1:** Baseline characteristics of the study population.

		DR group		P value
	Non-DR group	NPDR group	PDR group	
	n = 1,795,271	n = 178,051	n = 12,794	
**Age**, **years**	57.83 ± 12.29	62.37 ± 9.76	60.08 ± 9.42	
**Age**, **≥60 years**	838383 (46.7)	114480 (64.3)	7017 (54.85)	<0.0001
**Men**, **n (%)**	1080513 (60.19)	84527 (47.47)	6916 (54.06)	<0.0001
**Smoking**				<0.0001
Non-smoker	996540 (55.51)	120297 (67.56)	8187 (63.99)	
Former smoker	338029 (18.83)	32920 (18.49)	2493 (19.49)	
Current smoker	460702 (25.66)	24834 (13.95)	2114 (16.52)	
**Drinking**				<0.0001
Non	1024060 (57.04)	130829 (73.48)	9593 (74.98)	
Mild	616695 (34.35)	39519 (22.2)	2684 (20.98)	
Heavy	154516 (8.61)	7703 (4.33)	517 (4.04)	
**Regular physical activity**	859607 (47.88)	82652 (46.42)	5720 (44.71)	<0.0001
**Low income**	385203 (21.46)	38182 (21.44)	3008 (23.51)	<0.0001
**Hypertension**	1052010 (58.6)	121134 (68.03)	9429 (73.7)	<0.0001
**Dyslipidemia**	760547(42.36)	96741 (54.33)	6826 (53.35)	<0.0001
**Stroke**	43589 (2.43)	6445 (3.62)	545 (4.26)	<0.0001
**Heart disease**	98443 (5.48)	15255 (8.57)	894 (6.99)	<0.0001
**BMI**, **kg/m**^**2**^	25.14 ± 3.36	24.66 ± 3.19	24.16 ± 3.11	<0.0001
**Waist circumference**, **cm**	85.62 ± 8.55	84.93 ± 8.45	84.17 ± 8.34	<0.0001
**SBP**, **mmHg**	129.18 ± 15.64	128.32 ± 15.88	130.24 ± 17.05	<0.0001
**DBP**, **mmHg**	79.14 ± 10.09	76.73 ± 9.84	77.3 ± 10.38	<0.0001
**Fasting glucose**, **mg/dL**	143.15 ± 42.91	142.29 ± 49.81	154.21 ± 60.15	<0.0001
**Total cholesterol**, **mg/dL**	196.06 ± 41.5	182.93 ± 40.64	187.07 ± 44.14	<0.0001
**HDL**, **mg/dL**	51.24 ± 17.29	50.2 ± 16.95	49.58 ± 19.41	<0.0001
**GFR**, **ml/min/1**.**73 m**^**2**^	84.76 ± 35.96	79.23 ± 35.27	75.2 ± 33.75	<0.0001

Data are expressed as mean ± standard deviation or as number (percentage).

DR: diabetic retinopathy; ESRD: end-stage renal disease; NPDR: non-proliferative diabetic retinopathy; PDR: proliferative diabetic retinopathy.

### Incidence and risk of psoriasis among diabetic patients with DR and PDR

**Figure 2:**
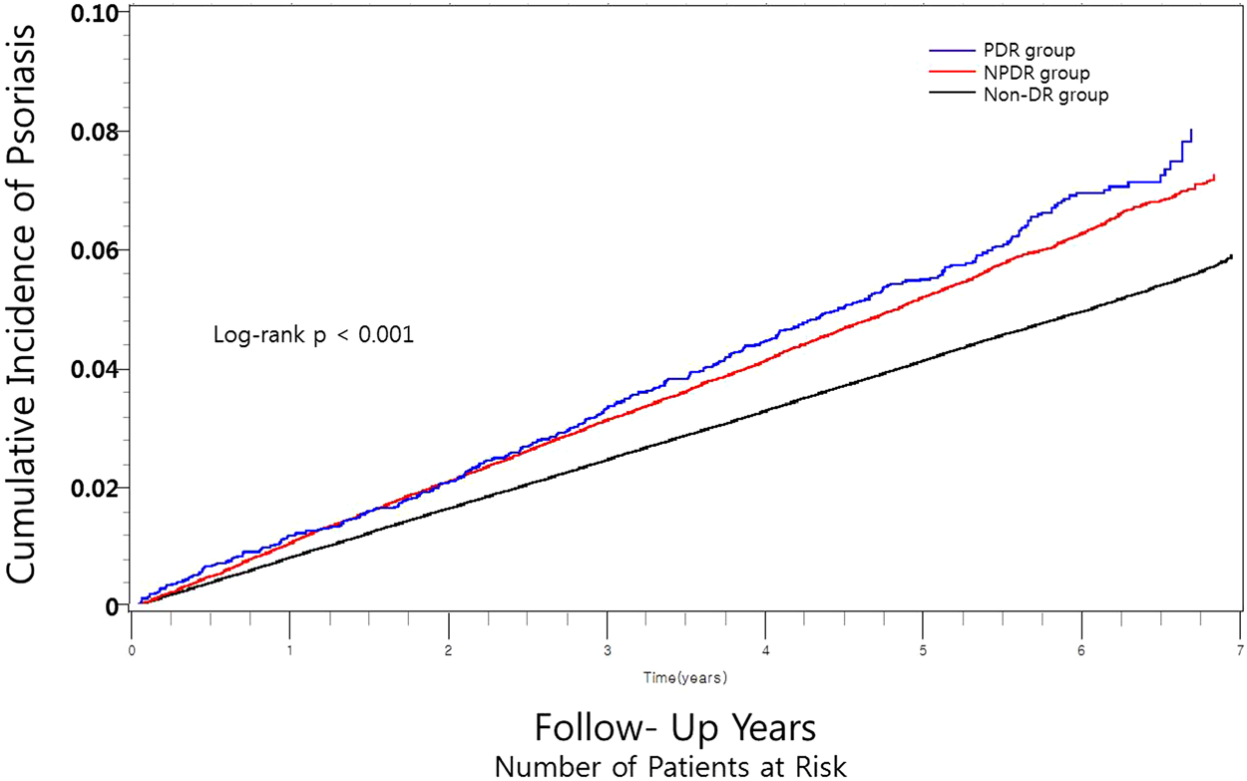
Cumulative incidence of psoriasis according to the presence and severity of diabetic retinopathy. DR = diabetic retinopathy; NPDR = non-proliferative diabetic retinopathy; PDR = proliferative diabetic retinopathy.

After a mean follow-up of 5.18 ± 0.05 years, 43,792 patients (2.20% of diabetic patients) developed psoriasis. Subjects with NPDR and PDR showed a tendency to suffer from psoriasis compared to subjects without DR (log-rank (overall) p < 0.001, Fig. 2). The incidence rates of psoriasis were 4.19, 5.31, and 5.74 per 1000 person-years in the non-DR, NPDR, and PDR groups, respectively (p for trend < 0.001). The severity of DR and the incidence of psoriasis were positively correlated and tended to increase.

In Table 2, after adjusting for age and gender (model 1), NPDR and PDR were significantly associated with the development of psoriasis. In model 2, after adjusting for confounding factors of age, gender, smoking, drinking, physical activity and income level, the incidence of psoriasis was significantly greater in the NPDR and PDR groups compared to the non-DR group. In model 3, after further adjustment for BMI, fasting glucose, hypertension, and dyslipidemia, NPDR group (HR 1.26, 95% CI 1.19–1.34) and PDR group (HR 1.35, 95% CI 1.22–1.50) had a significant risk of developing psoriasis compared to patients in the non-DR group. PDR was more strongly associated with development of psoriasis than was NPDR in both models 2 and 3.

**Table 2:** Incidence and risk of psoriasis among diabetic patients with NPDR and PDR.

Group	Psoriasis	Person-years	Psoriasis incidence (per 1000 person-years)	Model 1 HR (95% CI)	Model 2 HR (95% CI)	Model 3 HR (95% CI)
Non-DR	38450	9169672.8	4.19	1 (reference)	1 (reference)	1 (reference)
NPDR	4960	933894.42	5.31	1.23 (1.20,1.27)	1.23 (1.19,1.27)	1.26 (1.19,1.34)
PDR	382	66520.76	5.74	1.35 (1.22,1.50)	1.34 (1.21,1.48)	1.35 (1.22,1.50)

CI: confidence interval; DR: diabetic retinopathy; HR: hazard ratio; NPDR: non-proliferative diabetic retinopathy; PDR: proliferative diabetic retinopathy.

Model 1: adjusted for age and gender.

Model 2: adjusted for model 1 + smoking, drinking, regular physical activity, and income level.

Model 3: adjusted for model 2 + body mass index, fasting glucose, hypertension, and dyslipidemia.

### The impact of DR and PDR on psoriasis incidence according to presence of ESRD

As shown in Table 3, ESRD was significantly associated with incidence of psoriasis (Model 1: HR 2.66, 95% CI 2.36–2.99; Model 2: HR 2.59, 95% CI 2.30–2.91; Model 3: HR 2.59, 95% CI 2.30–2.91; p for trend < 0.001). We also analyzed the joint impact of ESRD and DR on the development of psoriasis. The risk of psoriasis was markedly increased in patients with both DR and ESRD (HR 2.98, 95% CI 2.48–3.59) compared to those with DR without ESRD (HR 1.22, 95% CI 1.19–1.26) (Model 2). Similarly, in Model 3, patients with both DR and ESRD had an increased risk of psoriasis (HR 3.00, 95% CI 2.50–3.61) compared to those with only DR (HR 1.22, 95% CI 1.19–3.61) (Table 3).

**Table 3:** Incidence and risk of psoriasis among diabetic patients with or without DR and ESRD.

	Psoriasis	Person-years	Psoriasis incidence (per 1000 person-years)	Model 1 HR (95% CI)	Model 2 HR (95% CI)	Model 3 HR (95% CI)
**Group**						
Non-ESRD	43514	10146096.17	4.29	1 (reference)	1 (reference)	1 (reference)
ESRD	278	23991.82	11.59	2.66 (2.36, 2.99)	2.59 (2.30, 2.91)	2.59 (2.30, 2.91)
**Group**						
Non-DR and Non-ESRD	38286	9154365.31	4.18	1 (reference)	1 (reference)	1 (reference)
Non-DR and ESRD	164	15307.50	10.71		2.47 (2.12, 2.88)	2.47 (2.11, 2.88)
DR and Non-ESRD	5228	991730.87	5.27		1.22 (1.19, 1.26)	1.22 (1.19, 1.26)
DR and ESRD	114	8684.32	13.13		2.98 (2.48, 3.59)	2.99 (2.49, 3.59)

CI: confidence interval; DR: diabetic retinopathy; ESRD: end-stage renal disease; HR: hazard ratio.

Model 1: adjusted for age and gender.

Model 2: adjusted for model 1 + smoking, drinking, regular physical activity, and income level.

Model 3: adjusted for model 2 + body mass index, fasting glucose, hypertension, and dyslipidemia.

## Discussion

This population-based study is the first to show an increased likelihood of psoriasis in DR patients. This research presented as follows: (1) the presence of NPDR and PDR was an risk factor of psoriasis onset in DM patients, and (2) ESRD was one of the predictors of psoriasis risk in diabetic patients and the presence of ESRD further increased the incidence of psoriasis in DR patients.

Some researchers have conducted a study on the relationship between psoriasis and DM, but the results are still inconclusive. Cohen *et al*. showed a significantly increased incidence of diabetes in patients with psoriasis after adjusting for age and sex (OR, 1.58, P < 0.001)^17^. Another study reported increased DM risk in psoriatic patients, and the risk increases with the duration and severity of psoriasis^18^. The results of Yeung *et al*. and Lønnberg *et al*. were consistent with previous studies showing that type 2 DM increases in psoriatic patients^19,20^. In a further meta-analysis, OR of DM risk in patient with mild psoriasis was 1.53 and OR of DM risk in patient with severe psoriasis was 1.97, respectively^21^. However, few studies have been conducted in the opposite direction. Jacob and Kostev reported that the risk of psoriasis in type 2 DM patients was higher than in controls (HR = 1.18, 95% CI: 1.08–1.29)^11^. Huerta *et al*. reported that there was no significant association between diabetes and psoriasis risk (OR, 0.7 [95% CI, 0.6–0.9])^10^. Meanwhile, previous studies haves shown that psoriasis, not including mild psoriasis, is an independent risk factor for chronic kidney disease (CKD) and ESRD events^22^. Chiu *et al*. showed that patients with psoriasis had a higher risk of CKD compared to controls^23^. Chi *et al*. found that patients with severe psoriasis had three times the risk of developing ESRD compared to patients without psoriasis^22^. However, there was no study of whether ESRD was a risk factor for psoriasis in diabetic patients.

DR and diabetic nephropathy commonly result from systemic microvascular impairment^24,25^. DR is one of the leading causes for visual impairment and diabetic nephropathy leads ESRD^24,26,27^. Microvascular complications of DM tend to increase with the prevalence, disease duration, or disease control of DM and have common risk factors of old age, hypertension, and high HbA1c level^16,24,28,29^. Psoriasis is considered an immune-mediated inflammatory skin disease. Psoriasis is also accompanied by vascular proliferation and inflammation^30^. This study showed the increased incidence of psoriasis in DM patients with microvascular complications such as DR and ESRD. Several potential mechanisms can explain the association between DR/ESRD and psoriasis. A previous report suggested that low-grade inflammation might provide a potential link between psoriasis and DM^31^. The association between psoriasis and DR/ESRD might be due to a chronic inflammatory environment. In particular, these effects might explain the increased risk of psoriasis in DR patients with ESRD. Elevated levels of TNF-α, a key player of angiogenesis, and other cytokines including IL-1 and IL-6 in psoriasis are associated with type 2 diabetes^32–34^. In addition, obesity as well as elevated levels of adipokines, such as leptin and adiponectin, are commonly found in psoriasis and type 2 DM^35–37^. Environmental factors such as smoking also affect both diseases^38,39^. Thus, inflammatory responses, adipokines, obesity, and environmental factors are commonalities that might explain the co-occurrence of these diseases.

There are some limitations of the present study. It was based on the ICD-10 code of claim data when defining DM as well as diabetic complications including DR and ESRD. Therefore, it was not possible to pinpoint whether the severity/duration of DM was related to psoriasis. For the same reason, this study could not analyze the effect of DR on severity of psoriasis. Meanwhile, the ‘non-informative censoring’ assumption for the Cox Proportional Hazards model is important, but since all patients were monitored until the end of the study in this study, the time until event distribution was independent of the time until censoring. However, the additional limitation is that time-dependent variables for the patient’s medical condition were not considered while tracking the risk of psoriasis. In addition, this study may not have sufficient follow-up time to assess the incidence of psoriasis in patients with DM complication. This study design included known confounding factors, but bias from unknown confounding factors could not be ruled out. However, we show consistent results in models that modulate various confounding factors.

Despite these limitations, we have found that DR is one of the risk factors for psoriasis, and development of psoriasis is related to the severity of DR. ESRD also had an additional independent factor for risk of psoriasis. In particular, in patients with diabetes, coexisted DR and ESRD have a synergistic effect on the development of psoriasis. Therefore, physicians should pay attention to the development of psoriasis in diabetic patients with complications.
